# Bridging Pediatric and Adult Rehabilitation Services for Young Adults With Childhood-Onset Disabilities: Evaluation of the LIFEspan Model of Transitional Care

**DOI:** 10.3389/fped.2021.728640

**Published:** 2021-09-17

**Authors:** Shauna Kingsnorth, Sally Lindsay, Joanne Maxwell, Yani Hamdani, Angela Colantonio, Jingqin Zhu, Mark Theodore Bayley, Colin Macarthur

**Affiliations:** ^1^Bloorview Research Institute, Toronto, ON, Canada; ^2^Holland Bloorview Kids Rehabilitation Hospital, Toronto, ON, Canada; ^3^Department of Occupational Science and Occupational Therapy, University of Toronto, Toronto, ON, Canada; ^4^Rehabilitation Sciences Institute, University of Toronto, Toronto, ON, Canada; ^5^Azrieli Adult Neurodevelopmental Centre, Centre for Addiction and Mental Health, Toronto, ON, Canada; ^6^Toronto Rehabilitation Institute, University Health Network, Toronto, ON, Canada; ^7^Canada Research Chair (Tier 1) in Traumatic Brain Injury in Underserved Populations, Canada Research Chair Program, Ottawa, ON, Canada; ^8^The Hospital for Sick Children, Toronto, ON, Canada; ^9^ICES, Toronto, ON, Canada; ^10^Division of Physical Medicine and Rehabilitation, Faculty of Medicine, University of Toronto, Toronto, ON, Canada; ^11^Child Health Evaluative Sciences, Hospital for Sick Children Research Institute, Toronto, ON, Canada; ^12^Institute of Health Policy, Management and Evaluation, University of Toronto, Toronto, ON, Canada

**Keywords:** continuity of care (COC), inter-agency partnership, pediatric healthcare providers, adult healthcare providers, Canada (MeSH), disability, healthcare utilization, transition to adult care

## Abstract

**Background:** LIFEspan (“Living Independently and Fully Engaged”) is a linked transition service model for youth and young adults with childhood-onset disabilities offered via an inter-agency partnership between two rehabilitation hospitals (one pediatric and one adult) in Toronto, Canada.

**Objective:** The objective was to evaluate healthcare outcomes (continuity of care and healthcare utilization) for clients enrolled in LIFEspan.

**Methods:** A prospective, longitudinal, observational mixed-method study design was used. The intervention group comprised youth with Acquired Brain Injury (ABI) and Cerebral Palsy (CP) enrolled in LIFEspan. A prospective comparison group comprised youth with Spina Bifida (SB) who received standard care. A retrospective comparison group comprised historical, disability-matched clients (with ABI and CP) discharged prior to model introduction. Medical charts were audited to determine continuity of care, i.e., whether study participants had at least one visit to an adult provider within 1 year post-discharge from the pediatric hospital. Secondary outcomes related to healthcare utilization were obtained from population-based, health service administrative datasets. Data were collected over a 3-year period: 2 years pre and 1 year post pediatric discharge. Rates were estimated per person-year. Fisher's Exact Test was used to examine differences between groups on the primary outcome, while repeated measures GEE Poisson regression was used to estimate rate ratios (post vs. pre) with 95% confidence intervals for the secondary outcomes.

**Results:** Prospective enrolment comprised 30 ABI, 48 CP, and 21 SB participants. Retrospective enrolment comprised 15 ABI and 18 CP participants. LIFEspan participants demonstrated significantly greater continuity of care (45% had engagement with adult services in the year following discharge at 18 years), compared to the prospective SB group (14%). Healthcare utilization data were inconsistent with no significant changes in frequency of physician office visits, emergency department visits, or hospitalizations for clients enrolled in LIFEspan in the year following discharge, compared to the 2 years prior to discharge.

**Conclusion:** Introduction of the LIFEspan model increased continuity of care, with successful transfer from pediatric to adult services for clients enrolled. Data on longer-term follow-up are recommended for greater understanding of the degree of adult engagement and influence of LIFEspan on healthcare utilization following transfer.

## Introduction

Advances in technology and healthcare practice have significantly increased survival rates for individuals with childhood-onset disabilities, with most now living well into adulthood (1, 2). In Canada, these young adults must transition from pediatric to adult healthcare systems at 18 years of age to manage their lifelong rehabilitation needs (3). In this transition, healthcare delivery should be a coordinated and uninterrupted provision of developmentally appropriate and comprehensive services (4). Transition from the pediatric to adult healthcare system is a complex process, however; and there is significant evidence that healthcare systems are not yet designed to effectively meet lifelong rehabilitation needs (2, 5, 6). Adolescents with childhood-onset disabilities are often significantly under-serviced as young adults, while some receive no care at all (2). Recurring themes related to barriers experienced during this transition include poor links between pediatric and adult rehabilitation services, insurance coverage restrictions, inadequate access to adult providers because of narrow criteria focused on adult-onset disabilities, and a general lack of specialized training and expertise related to aging with a disability or chronic illness (7, 8). In the absence of adequate services, health issues can go unmonitored and/or untreated, increasing the risk of preventable complications, an inappropriate reliance on emergency health services, and increased hospital admissions (9, 10).

Responding to calls for action, there has been a surge in transition models and activities, with a growing body of literature seeking to understand “what works” for young adults with specialized healthcare needs. Numerous systematic reviews of transition interventions that address transition planning, transfer assistance, and integration into adult services demonstrate positive outcomes from such interventions related to population health, continuity or adherence to care, healthcare utilization, and satisfaction with care (11–13). Examples of transition models range from dedicated young adult clinics within adult services, pediatric clinics with structured processes, and “bridge programs” where components of pediatric and adult care are included, e.g., dual visit models where adult and pediatric providers are both present (12, 14). An additional distinguishing feature is whether transition models are built around a dedicated facilitator, or are based on a multi-disciplinary team approach (15). To a large extent, transition services have predominantly focused on chronic illness, such as diabetes, heart disease, kidney transplant and juvenile idiopathic arthritis (11, 13, 16, 17). In comparison, fewer studies have examined service models and outcomes for young people with childhood-onset disabilities (18–21).

The LETS study (“Longitudinal Evaluation of Transition Services”) aims to contribute to this emerging evidence base by examining healthcare outcomes for youth and young adults with childhood-onset disabilities enrolled in a linked transition service delivery model (22). In Toronto, Ontario, two hospitals partnered to develop LIFEspan (“Living Independently and Fully Engaged”). This inter-agency collaboration was designed to formally link pediatric and adult rehabilitation services for youth with a diagnosis of childhood Acquired Brain Injury (ABI) or Cerebral Palsy (CP). The linkage was reflected in cross-appointed staff, a multi-disciplinary allied health team and standardized care processes, supporting a 2-year transition preparation at the pediatric hospital followed by a coordinated transfer to the adult hospital for ongoing care. The model reflects key principles outlined in a Canadian national guideline for transitioning from pediatric to adult care (23). Prior publications have reported on healthcare provider perspectives and experiences within the LIFEspan model and perceived successes related to enhanced transition preparation (18, 24, 25). The objective of this study was to quantitatively examine the impact of the LIFEspan model on healthcare outcomes, specifically continuity of care and healthcare utilization. It was hypothesized *a priori* that clients in the LIFEspan model would have better continuity of care and a reduced reliance on emergency services for their care, compared with prospective, and historical controls.

## Methods

### Study Design

The LETS Study was a prospective, longitudinal, observational mixed-method study evaluating the LIFEspan model of transition care. This study was completed before the COVID-19 pandemic. The full protocol has previously been published (22). This manuscript addresses the primary outcome of continuity of care through medical record audit; as well as secondary outcomes of healthcare utilization (physician office visits, emergency department visits, and hospitalization) through health services administrative dataset review. Diagnostic study groups included three childhood-onset neurological conditions: Acquired Brain Injury (ABI), Cerebral Palsy (CP) and Spina Bifida (SB).

### Intervention

#### LIFEspan

The LIFEspan program is a coordinated, client-centered model of linked healthcare offered through a collaborative partnership between a pediatric rehabilitation centre (Holland Bloorview Kids Rehabilitation Hospital, Toronto, Canada) and an adult rehabilitation centre (Toronto Rehabilitation Institute, Toronto, Canada). Supported by multi-disciplinary healthcare teams, clients engage in a 2-year period of healthcare transition preparation from 16 to 18 years of age and are seen 2–4 times depending on individual need. During this period, they are medically followed (by pediatric physician/ambulatory care nurse or nurse practitioner) and work on transition-related goals (e.g., social participation, community involvement) with support from a youth facilitator, life skills facilitator and/or social worker. This preparation is followed by discharge from the pediatric hospital and a coordinated transfer to adult care around the age of 18 led by cross-appointed LIFEspan staff – nurse practitioner, youth facilitator and life skills facilitator – in the context of a formal linkage between the two rehabilitation centres. In addition to the cross-appointed roles, the adult LIFEspan team includes a physiatrist, social worker, occupational therapist, physiotherapist and speech language pathologist. Continued transition supports in the adult clinic focus on interventions to address goals set by the young adults and their families, and establishing linkages to community services, and allied and primary care resources. Details of the LIFEspan model have previously been described (24, 25).

At the time of this study, two pediatric clinics at Holland Bloorview serving clients with ABI and CP streamed into a single adult ABI/CP clinic at Toronto Rehabilitation Institute. Alternatively, clients could opt out of the LIFEspan service model pathway and make other choices for adult care provision.

#### Standard of Care

The Spina Bifida clinic at Holland Bloorview is supported by a multi-disciplinary team including nursing, occupational therapy, social work, psychology, physiotherapy and a life skills facilitator, as well as a pediatric physician (neonatology and developmental pediatrics), and consulting specialists in urology and orthopedics. Clients are seen annually, with intervention and consultation services as required. Around 18 years of age, these clients were referred to a local tertiary hospital (Sunnybrook Health Sciences Centre, Toronto, Canada) or other adult care provision of their choosing on discharge.

### Recruitment and Sample

Study participants were recruited from Holland Bloorview; a large, urban, pediatric academic health sciences center supporting inpatient and outpatient rehabilitation needs of children and youth from birth to 18 years of age with physical disabilities and complex medical needs. Institutional policy mandated that all clients with ABI and CP received LIFEspan services. Thus, eligible participants for the “prospective” arm of the study were clients 16 years of age with a diagnosis of ABI or CP and enrolled in the LIFEspan model.

The LIFEspan program had no “waitlist” of clients from which to select a comparison group as all eligible clients were enrolled in the program. Therefore, alternative comparison groups were identified. First, clients 16 years of age in the Spina Bifida clinic at Holland Bloorview were selected as a prospective comparison group receiving “standard of care.” In general, people with SB face the same challenges as those with ABI and CP with respect to complexity of care, the need for ongoing monitoring, and holistic support to maximize their health and wellness, social participation and community involvement. Individuals with SB also experience the same gaps in obtaining adult health services with the attendant consequences (5, 26). There are often, however, significant differences between these diagnostic groups with respect to baseline health status, clinical management, and health care utilization.

In addition to the prospective SB comparison group, data were also collected on a “retrospective” historical comparison group, consisting of past clients with ABI and CP at Holland Bloorview who had transitioned to adult care services without participating in the LIFEspan model. Data were collected over the same 3-year period for the comparison groups, i.e., for each client from 16 to 19 years of age.

Recruitment lists were generated by health data services based on date of birth (i.e., 16 years of age at enrolment) and diagnosis, with 88 ABI, 128 CP, and 43 SB clients identified for the prospective arm; and 61 ABI and 71 CP former clients for the retrospective arm. Prospective participants were recruited in-person. Retrospective participants were recruited via mailed information packages and follow-up phone calls. All participants provided written informed consent or written informed assent with parental/guardian consent. Ethics approval for the study was granted by Holland Bloorview Kids Rehabilitation Hospital, Toronto Rehabilitation Institute and Sunnybrook Health Sciences Centre.

### Outcome Measures

#### Demographic Factors

Demographic and clinical information were collected on participants: sex, ethnicity and diagnostic details. The general health of prospective participants was self-rated using a global health question [“In general, would you say your health is: excellent (5), very good (4), good (3), fair (2) or poor (1)?”] (27).

#### Primary Outcome Measure

The primary outcome of interest was the maintenance of *continuous care*, given that the published literature suggests that the core indicator of transition success is the minimization of loss of patients from pediatric discharge to adult follow-up (28). Lotstein et al. defines continuity of care as “ongoing access to age- and disease-appropriate health care” (29). In a systematic review of continuity of care during transfer to adult services, attendance at an adult clinic visit and/or time between last pediatric clinic visit and first adult clinic visit were the most common measures of engagement in adult care (17).

Medical charts at the adult hospitals (Toronto Rehabilitation and Sunnybrook) were audited to determine whether prospective participants had at least one visit to an adult provider within 1 year post-discharge (at around 18 years) from the pediatric rehabilitation hospital.

#### Secondary Outcome Measures

Healthcare utilization data were also collected on physician visits, emergency department visits, and hospitalizations determined from population-based, health services administrative datasets held by ICES, using participants' unique Ontario Health Insurance Plan (OHIP) number. ICES is an independent, non-profit research institute funded by an annual grant from the Ontario Ministry of Health (MOH) and the Ministry of Long-Term Care (MLTC). As a prescribed entity under Ontario's privacy legislation, ICES is authorized to collect and use healthcare data for the purposes of health system analysis, evaluation, and decision support. Secure access to these data is governed by policies and procedures that are approved by the Information and Privacy Commissioner of Ontario. These datasets were linked using unique encoded identifiers and analyzed at ICES.

### Statistical Analyses

For the primary outcome, Fisher's Exact Test was used to test for differences in the proportion of clients with continuous care between the prospective LIFEspan group and the prospective SB comparison group. For the secondary outcomes, the frequency of physician office visits, emergency department visits, and hospitalizations over the 3-year period of study were calculated per person-year. Each of the three groups—the LIFEspan group, the prospective SB group, and the retrospective historical comparison group—acted as their own control. In other words, the frequency of healthcare utilization in the year following discharge from the pediatric rehabilitation hospital (post) was compared with healthcare utilization in the 2 years prior to discharge (pre). Repeated measures GEE Poisson regression was used to estimate rate ratios (post vs. pre) along with 95% confidence intervals for the prospective LIFEspan group, the prospective SB group, and the retrospective historical (ABI+CP) group.

## Results

### Participants

In total, 132 clients participated in the study: the prospective arm included 78 clients in the LIFEspan group (30 ABI and 48 CP) and 21 SB clients in the prospective comparison group. The retrospective “historical” comparison group consisted of 33 participants (15 ABI and 18 CP). Recruitment rates based on eligible clients were 30% for the prospective arm and 25% for the retrospective arm.

Table 1 shows the demographic characteristics of the participants in the prospective arm; most were male (64%) and born in Canada (88%). With respect to ratings of global health, both ABI and CP participants reported a median rating of 4 (“very good”) and SB reported a median rating of 3 (“good”).

**Table 1:** Clinical and demographic characteristics of (prospective) participants at study enrolment (16 years of age).

	**ABI**	**CP**	**SB**
	***N* (%)**	***N* (%)**	***N* (%)**
**Sex**			
Male	19 (63)	30 (62)	10 (48)
Female	11 (37)	18 (38)	11 (52)
**Diagnostic details**	Injury: 20 (67)	GMCFS I: 12 (25)	Lipomyelomingocele: 17 (81)
	Medical: 10 (33)	GMCFS II: 5 (10)	Myelomeningocele: 2 (9.5)
		GMCFS III: 8 (17)	Not reported: 2 (9.5)
		GMCFS IV: 4 (8)	
		GMCFS V: 10 (21)	
		Other/Not reported: 9 (19)	
**Canadian born** ^*^	17 (81)	32 (88)	14 (93)
**Ethnicity** ^*^			
Caucasian	11 (52)	17 (47)	8 (53)
Black	6 (29)	7 (19)	2 (13)
Asian	2 (9)	6 (17)	4 (27)
Other	1 (5)	2 (6)	1 (7)
Prefer not to answer	1 (5)	4 (11)	0 (0)
**Global health rating** ^*^	Median: 4	Median: 4	Median: 3
	Min: 1	Min: 2	Min: 2
	Max: 5	Max: 5	Max: 5

* *Data not provided for 9 ABI, 12 CP and 6 SBI*.

### Primary Outcome Measure

Of the 78 clients enrolled in LIFEspan, 35 (45%) had formal engagement with healthcare services in the adult hospitals within 1 year post-discharge (i.e., after 18 years of age), compared with only three of 21 (14%) SB clients (*p* = 0.012) during this window.

### Secondary Outcome Measures

Of note, several participants in the prospective arm (5 ABI, 2 SB) did not consent to providing OHIP numbers and therefore population-based, health services administrative data could not be collected on these participants. Physician office visits, emergency department (ED) visits, and hospitalization data are presented in Table 2.

**Table 2:** Secondary outcomes: physician office visits, emergency department (ED) visits, and hospitalizations by study groups.

	**Pre** ^*^ **events**	**Rate/person-year**	**Post** ^**∧**^ **events**	**Rate/person-year**	**Rate ratio** ^**#**^ **(post vs. pre)**	**95% CI**
**Office visits**						
LIFEspan	716	4.90	340	4.66	0.95	0.77–1.17
SB	261	6.87	148	7.79	1.13	0.86–1.50
Historical	503	7.62	197	5.97	0.78	0.59–1.04
**ED visits**						
Lifespan	39	0.27	31	0.42	1.59	0.98–2.58
SB	30	0.79	12	0.63	0.80	0.41–1.54
Historical	25	0.38	10	0.30	0.80	0.36–1.76
**Hospitalizations**						
LIFEspan	10	0.07	3	0.04	0.60	0.17–2.16
SB	13	0.34	1	0.05	0.15	0.02–1.38
Historical	8	0.12	0	0	N/A	

* *Two years prior to discharge*.

∧ *One year post-discharge*.

# *Repeated Measures GEE Poisson Regression Analysis*.

#### Physician Office Visits

On average, participants in the LIFEspan group had 4.82 physician office visits per person-year (1,056 total over the 3 years). The rate ratio for the LIFEspan group (post vs. pre) was 0.95 (95% CI: 0.77–1.17), *p* = 0.626. The prospective SB group had 7.18 physician office visits per person-year (409 total over 3 years), with a rate ratio (post vs. pre) of 1.13 (95% CI: 0.86–1.50), *p* = 0.373. The retrospective historical (ABI+CP) group had 7.07 physician office visits per person-year (700 total over the 3 years), with a rate ratio (post vs. pre) of 0.78 (95% CI: 0.59–1.04), *p* = 0.096.

In summary, the LIFEspan group had fewer physician office visits in the year after discharge from Holland Bloorview, compared with pre-discharge; however, this difference was trivial and not statistically significant. Likewise, there were no significant differences in physician office visits post- vs. pre- discharge for the prospective SB group and the retrospective historical (ABI+CP) group.

#### Emergency Department Visits

On average, participants in the LIFEspan group had 0.32 ED visits per person-year (70 total over the 3 years). The rate ratio for the LIFEspan group (post vs. pre) was 1.59 (95% CI: 0.98–2.58), *p* = 0.060. The prospective SB group had 0.74 ED visits per person-year (42 total over 3 years), with a rate ratio (post vs. pre) of 0.80 (95% CI: 0.41–1.54), *p* = 0.505. The retrospective historical (ABI+CP) group had 0.35 ED visits per person-year (35 in total over the 3 years), with a rate ratio (post vs. pre) of 0.80 (95% CI: 0.36–1.76), *p* = 0.580.

In summary, the LIFEspan group had more ED visits in the year after discharge from Holland Bloorview, compared with pre-discharge; however, this difference was modest and not statistically significant. Similar findings, i.e., no significant difference in ED visits post- vs. pre- discharge for the prospective SB group and the retrospective historical (ABI+CP) group were also noted.

#### Hospitalizations

On average, participants in the LIFEspan group had 0.06 hospitalizations per person-year (13 total over the 3 years). The rate ratio for the LIFEspan group (post vs. pre) was 0.60 (95% CI: 0.17–2.16), *p* = 0.434. The prospective SB group had 0.25 hospitalizations per person-year (14 total over 3 years), with a rate ratio (post vs. pre) of 0.15 (95% CI: 0.02–1.38), *p* = 0.095. The retrospective historical (ABI+CP) group had 0.08 hospitalizations per person-year (8 in total over the 3 years). There were no hospitalizations, however, in this group in the year following discharge, which made calculation of a post vs. pre rate ratio unfeasible.

In summary, the LIFEspan group had fewer hospitalizations in the year following discharge from Holland Bloorview, compared with pre-discharge; however, this difference was modest and not statistically significant. Likewise, there was no significant difference in hospitalizations post- vs. pre-discharge for the prospective SB group.

## Discussion

Introduction of the LIFEspan model of linked transitional care led to increased continuity of care (as measured by engagement with adult services within 1 year of pediatric discharge) for clients with ABI and CP, compared to clients with SB who were not enrolled in the model. Healthcare utilization data were inconsistent and showed no significant changes in physician office visits, ED visits, or hospitalizations for clients in the LIFEspan program in the year following discharge from the pediatric rehabilitation hospital, compared to the 2 years prior to discharge. Previously published Canadian research on childhood disabilities shows high rates of urgent care use by these specific clinical populations (26, 30).

This finding of successful transfer from pediatric to adult services aligns with previous qualitative evaluations of the LIFEspan model, based on provider reflections of increasing caseloads in the adult clinic following model launch (25) and positive transition experiences described by clients with ABI and their parents (18). In contrast to the comparison SB clinic that offered “standard of care,” specific design features of the LIFEspan model may have fostered relational continuity through the cross-appointed staff roles as well as management continuity through the formal partnership between a pediatric and an adult rehabilitation hospital (31).

From a service delivery perspective, several studies have examined a variety of transition outcomes for “bridging” models, also described in the literature as “integrated” (32), “concurrent” (33), “intra-agency” (34), “co-located” (35) or “inter-agency” (19). For example, Harden et al. described an integrated joint multi-disciplinary pediatric-adult transition clinic and care pathway for youth with kidney failure (32). In this approach, patients were seen jointly by two teams from 15 to 18 years of age and then transferred by the age of 18 years to adult services. Enhanced engagement with healthcare providers and improved adherence to medication were noted, leading to reduced transplant failure rates compared with historical controls (32). Semalulu et al. described a similar joint transition program for youth with juvenile idiopathic arthritis and systematic lupus erythematosus (33). From 14 to 18 years of age, patients had concurrent pediatric and adult rheumatologist visits as part of a multi-disciplinary pediatric team; provision of care by the adult rheumatologist continued till 22 years of age following transfer to the young adult clinic. The study described trust as a key component of transition preparedness, with favorable perceptions of patient-provider relationships increasing with age (33). Van Pelt et al. conducted a longitudinal observational study of an intra-agency nurse facilitator model for youth with juvenile idiopathic arthritis with pediatric and adult clinics within the same medical centre (34). Patterns of drop-out during the transfer window of 16–18 years relative to other age windows—pediatric (10–13 years) and adult (18–27 years)—were examined. Relative to the comparison windows, drop-out rates were higher for the facilitator model during this period of upheaval, but still lower than rates noted in the literature in the absence of structured transition processes (34).

Nolan et al. further describe a co-location model distinguished by overlap in pediatric and adult care for young adults with sickle cell disease (35). In the model, young adults (18–25 years) were seen by an adult internist, in addition to a cross-appointed pediatric hematologist and a pediatric transition nurse coordinator, in monthly clinics within the adult setting. Nursing transition case management began at age 17 years in the pediatric setting. Continuity of care was maintained after transfer for the 59 participants enrolled in the model (35). Specific to childhood-onset disability, Lindsay et al. examined an inter-agency transition model for spina bifida (19, 36). Analogous to LIFEspan, the model comprised a formal linkage between a pediatric and an adult hospital, and included a cross-appointed nurse practitioner and life skills coach as members of the transition teams at each site (19, 36). Care experiences were examined qualitatively for parents, youth and young adults with SB (14–21 years) at varying stages of healthcare transfer, relative to a cohort that had transitioned prior to model introduction. Whilst few of the intervention group had transferred out of pediatric care, experiential narratives described enhanced perceptions of support related to accessing adult care. Cross-sectoral linkages were identified as required in the model to fully address extensive social, educational and vocational needs (19).

Our study had several strengths. First, the use of comparison groups, contemporaneous and historical, allowed a direct comparison of healthcare outcomes across time and clinical conditions. Second, there was minimal loss to follow-up of participants. We also had a diverse sample in terms of race and ethnicity. Last, data were extracted from an established, population-based, administrative data source. With respect to study limitations, many potentially eligible pediatric clients were not interested in participating in the study; a lack of data made comparison of those who agreed to participate and those who did not unfeasible. In addition, proficiency in English was an inclusion criterion, which meant that the experiences of clients from other cultural and linguistic backgrounds were not captured. Medical records and administrative databases may also have inherent limitations, depending on the accuracy and completeness of information collected. Last, the evaluation of healthcare utilization was limited by small sample sizes, relatively few events, and a short follow-up period of 1 year. These limitations may explain the inconsistent and statistically non-significant results related to healthcare utilization. Our findings on continuity of care, however, are consistent with previous systematic reviews that have examined continuity of care following participation in structured healthcare transition processes (11, 13). We believe our study is particularly important, because of its emphasis on childhood-onset disabilities, given the previous predominant focus of evaluation studies on chronic illness (11–13, 16, 17).

Taken collectively, the current literature suggests that bridging models, such as LIFEspan, are particularly relevant for clients with childhood-onset disabilities, for whom multidisciplinary clinics are ideal for lifelong care (35). This necessity was highlighted in a 2016 survey of 11 nationally recognized US pediatric multi-disciplinary CP clinics. Survey respondents identified the limited number of adult providers willing to accept CP patients, concerns about the level of care in the adult healthcare system, and lack of financial resources as significant barriers that remained very “real and problematic.” Of all the participating clinics, only one had transitioned 100% of its clients to adult providers by 22 years of age (37).

## Conclusions

Clients who participated in the LIFEspan model were more likely to be *linked* to adult healthcare services following discharge from the pediatric hospital. Not all participants in the LIFEspan model were engaged with the adult healthcare system 1 year after discharge; however, the program appears to foster and enhance continuity of care outcomes for young adults with childhood-onset disabilities as they navigate the pediatric and adult service divide. With regards to future research, longer-term follow-up would provide a more in-depth understanding of the degree of adult engagement (specifically retention in the system) as well as long-term impacts on healthcare utilization. In this context, evaluation of outcomes three or more years after transition to adult care has been suggested (3, 37).

## Data Availability Statement

The datasets presented in this article are not readily available because data from this study are held securely in coded form at ICES. While legal data sharing agreements between ICES and data providers (e.g., healthcare organizations and government) prohibit ICES from making the dataset publicly available, access may be granted to those who meet pre-specified criteria for confidential access, available at www.ices.on.ca/DAS. The full dataset creation plan and underlying analytic code are available from the authors upon request, understanding that the computer programs may rely upon coding templates or macros that are unique to ICES and are therefore either inaccessible or may require modification. Requests to access the datasets should be directed to das@ices.on.ca.

## Ethics Statement

The study protocol was reviewed and approved by the Registered Ethics Board at each of the partnering hospitals: Holland Bloorview Research Ethics Board (Approval #09-036), University Health Network Research Ethics Board (Approval #10-009); and Sunnybrook Research Institute Ethics Office (Approval #251-2011). Written informed consent to participate was provided by participants, or assent to participate with consent by the participants' legal guardian/parent. The LETS Study is registered as a clinical trial: ID NCT00975338 with information available at www.clinicaltrials.gov.

## Author Contributions

SK, CM, MB, JM, SL, YH, and AC conceived the study design, obtained funding and contributed to execution of the project. SK, CM, and JZ performed data analysis and interpretation. SK and CM drafted the initial manuscript. SL, JM, YH, AC, JZ, and MB critically revised and approved the final manuscript. All authors contributed to the article and approved the submitted version.

## Funding

Funding for the LETS study was provided by the Ontario Neurotrauma Foundation (ONF), Award No. 2008-ABI-LSMODEL-706, and in part by the Canada Research Chairs Program. This study was also supported by ICES, which was funded by an annual grant from the Ontario Ministry of Health (MOH) and the Ministry of Long-Term Care (MLTC). Parts of this material are based on data and/or information compiled and provided by CIHI. The analyses, conclusions, opinions and statements expressed herein are solely those of the authors and do not reflect those of the funding or data sources; no endorsement is intended or should be inferred.

## Conflict of Interest

The authors declare that the research was conducted in the absence of any commercial or financial relationships that could be construed as a potential conflict of interest.

## Publisher's Note

All claims expressed in this article are solely those of the authors and do not necessarily represent those of their affiliated organizations, or those of the publisher, the editors and the reviewers. Any product that may be evaluated in this article, or claim that may be made by its manufacturer, is not guaranteed or endorsed by the publisher.

## Abbreviations

ABI: acquired brain injury
CP: cerebral palsy
ED: emergency department
SB: spina bifida.
